# Correlates of intention to screen for cervical cancer among adult women in Kyotera District, Central Uganda: a community based cross-sectional study

**DOI:** 10.1186/s12905-024-03129-5

**Published:** 2024-05-18

**Authors:** Arthur Kiconco, Richard Kabanda, Anguzu Ronald, Kirsten M. M. Beyer, Steven A. John

**Affiliations:** 1 Institute for Health and Equity, Medical College of Wisconsin, Milwaukee, WI 53226 USA; 2 Uganda Martyrs University, Nkozi, Uganda; 3 Ministry of Health, Kampala, Uganda; 4 Department of Psychiatry and Behavioral Medicine, Medical College of Wisconsin, Milwaukee, WI 53226 USA

**Keywords:** Intention, Cervical cancer screening, Knowledge, Uganda, Health information source

## Abstract

**Introduction:**

Cervical cancer continues to pose a major public health challenge in low-income countries. Cervical cancer screening programs enable early detection and effectively reduce the incidence of cervical cancer as well as late-stage diagnosis and mortality. However, screening uptake remains suboptimal in Uganda. This study assessed correlates of intention to screen for cervical cancer among women in the Kyotera district of Central Uganda.

**Methods:**

We analyzed cross-sectional data collected to determine the effectiveness of community audio towers (CATs) as a modality of health communication to support cervical cancer prevention. Women (*n* = 430) aged 21–60 years without a prior history of cervical cancer screening were surveyed about demographics, sources of health information and cervical cancer screening intentions in 2020. We used generalized linear modelling with modified Poisson regression and backwards variable elimination to identify adjusted prevalence ratios and 95% confidence intervals (CI) to determine factors associated with intention to screen for cervical cancer.

**Results:**

Half (50.2%) of the participants had intentions to screen for cervical cancer within twelve months and 26.5% had moderate knowledge about cervical cancer. Nearly half (46.0%) considered themselves at risk of cervical cancer. Compared to residents who primarily received their health information from social media and radio, participants who received health information primarily from CATs (aPR:0.64, 95% CI:0.52–0.80, *p* < 0.001) and TV (aPR:0.52, 95% CI:0.34–0.82, *p* = 0.005) had a lower prevalence of intention to screen for cervical cancer. The prevalence of intentions to screen for cervical cancer in twelve months was higher among those resided in town councils (aPR:1.44, 95% CI:1.12–1.86, *p* = 0.004) compared to rural areas, and higher among those who considered themselves to be at risk of cervical cancer (aPR:1.74, 95% CI:1.28–2.36, *p* < 0.001) compared to those who did not.

**Conclusions:**

We found suboptimal prevalence of intentions to screen for cervical cancer among women in central Uganda. Additional research and implementation projects are needed to increase cervical cancer screening. Targeting risk perceptions and behavioral approaches to increase intentions could be effective in future intervention work. Based on urban-rural differences, additional work is needed to support equitable sharing of information to support cancer prevention messaging; CATs and TV may best help reach those with lower intentions to screen based on our research.

## Background

Cervical cancer continues to pose a major global public health burden, with over 340,000 deaths annually [1]; projections estimate this number increasing to 400,000 annual deaths by 2030 [2]. Cervical cancer is the fourth most common cancer among women globally [3], with an estimated 604,127 new cases of cervical cancer in 2020 [1] and an anticipated increase to 700,000 by 2030 [4]. Cervical cancer is among the common human papillomavirus (HPV)-related diseases, with nearly all cases of cervical cancer attributable to HPV infection; specifically, HPV types 16 and 18 are known to cause 70% of cervical cancers and precancerous cervical lesions [5–7].

There are significant socioeconomic disparities in cervical cancer incidence rates, with national rates increasing as the Human Development Index (HDI) decreases; the poor, especially in low- and middle-income countries (LMICs), shoulder the largest disease burden [1]. The highest cervical cancer incidence occurs in Africa, followed by Latin America, Asia, and Melanesia. Within sub-Saharan Africa, the 2020 age-adjusted incidence rate for cervical cancer was highest in eastern Africa, estimated at 40 cases per 100,000 women-years [1]. The 2023 age-adjusted incidence and mortality rates for Uganda were 56.2 and 41.4 respectively [8]. In the same year, the annual estimates indicated that 6,959 women were diagnosed with cervical cancer and 4,607 died from the disease—making it the first most frequent cancer among women in Uganda [8].

Despite disparities in cervical cancer incidence rates, resources for prevention, diagnosis and treatment are limited in most LMICs [9, 10]. Although preventable and curable if identified at an early stage, cervical cancer remains a top cancer killer of women in low-resource settings [11]. The HIV/AIDS epidemic is also believed to exasperate high rates of cervical cancer incidence and mortality, as the risk of development, progression, and recurrence of HPV-induced cervical precursor lesions and cervical cancer are higher among women living with HIV (WLHIV) [12–16]. Despite reductions in HIV new infections in Uganda, the HIV prevalence remains high at 7.2% among women compared to 4.3% among men [17].

The World Health Organization (WHO) Global Cervical Cancer Elimination Initiative (GCCEI) aims to reduce incidence below a threshold of 4 cases per 100,000 women-years in every country [2]. Cervical cancer is the number one cause of cancer-related deaths among women in Uganda [18], and the WHO estimates approximately 3,915 Ugandan women were diagnosed with cervical cancer and 2,160 died from the disease in 2014 [19]. In Uganda, cervical cancer screening guidelines recommend visual inspection of the cervix with acetic acid (VIA) annually for women living with HIV and every 3 years for those HIV-negative [20].

Cervical cancer screening programs enable the detection of cervical lesions before they become cancerous, which can effectively reduce the incidence of cervical cancer by 75–90% [21, 22]. Screening also results in earlier detection of cancer, improving prognosis among those diagnosed and treated. As such, population-based cervical cancer screening programs are effective in reducing cervical cancer mortality [23, 24]. Despite these statistics, only a small percentage (estimated at 19%) of women have been screened for cervical cancer in LMICs, compared to 63% in high-income countries [25]. In Uganda, it is estimated that the percentage of women who had ever screened for cervical cancer ranged from 9 to 10% and only about 7.5% had screened in the last 5 years in 2023 [8].

Further, researchers have previously attributed low cervical cancer screening uptake to a number of key factors, including limited resources required for successful screening programs [25, 26], cervical cancer knowledge gaps [27–30], fear of positive diagnosis [31], and lower risk perception and negative attitudes [32]. The SARS-CoV-2 (i.e., COVID-19) pandemic is also believed to have led to delays in diagnosis and treatment due closures of health facilities, disruptions in access due to loss of insurance as people were laid off from work, and fear of COVID-19 exposure by those eligible for screening and care [33]. Most cervical cancer prevention programs aimed at increasing screening uptake usually focus on modifiable contextual factors such as knowledge, women’s intentions, and service availability, among others. However, few studies have assessed correlates of intention to screen for cervical cancer. As such, we assessed correlates of intention to screen for cervical cancer among adult women in Kyotera District, Central Uganda.

## Methods

### Study design

This was a cross-sectional analytical study based on secondary analysis of data collected at baseline for a study to determine the efficacy of community audio towers (CATs) as a health communication channel used in the prevention of cervical cancer in rural communities in Uganda [34]. The primary study was carried out between March and June 2020. It compared the use of CATs to disseminate messages on cervical cancer versus other health communication channels and cervical cancer screening among women aged 21 to 60 years. This analysis focused on data collected at baseline, prior to the use of the CATs for dissemination of cervical cancer-prevention messaging.

### Study setting

#### Study area

This study was carried out in Kyotera district, located in the south-central region, southwest of Kampala Capital City in Uganda. Kyotera District headquarters are approximately 182 km from Kampala and forty-seven kilometers from Masaka City. Kyotera District was created from Rakai District in the year 2015 by an Act of Parliament but started operating as an independent district and local government on July 1, 2017, with two counties of Kakuuto and Kyotera. The district is primarily rural and borders with Kalangala, Masaka, Rakai, and Lwengo districts in Uganda and the Missenyi district in the south, which is in the Kagera region of Republic of Tanzania.

Kyotera District was part of Rakai where the first case of HIV/AIDS in Uganda was discovered at the Uganda-Tanzania border of Mutukula [35]. The district is known for its high HIV prevalence, currently standing at 11.1% [36]. There is a known link between HIV/AIDS and cancers, including cancer of the cervix, which shares similar risk factors. Although there are no disaggregated data showing the district prevalence of cervical cancer, the prevalence of cancer of the cervix is likely high in Kyotera.

#### Inclusion and exclusion criteria

The study population consisted of women aged 21–60 years living in Kyotera district. To be eligible, participants were required to: (1) be aged 21–60 years; (2) have lived in Kyotera for at least 3 months; and (3) have direct access to information as narrow-casted from CATs. Participants were excluded if (1) they had previously screened for cervical cancer in the past three years or one year for those LHIV; and (2) intended to relocate in the proceeding 16 weeks at the time of the survey. All participants consented to participate in this study.

### Sample size and sampling

#### Sample size

The sample size for the parent study was 480 participants. Each cluster (village) had sixty participants, and eight clusters were included. Fifty (50) of the participants had screened in the previous three years and were excluded from this sample; thus, the final sample for analysis was 430 participants.

#### Sampling and recruitment procedures

The initial process of sampling was based on the composition of Kyotera district in terms of counties. Kyotera has two counties, and each of these forms a health subdistrict. Recruitment of participants from clustered villages was done by systematic sampling from a list of households registered by community health workers to have the targeted age group. Where households had more than one eligible participant, only one was sampled and the lottery method was used to select one.

### Study variables

#### Dependent variable

The dependent variable for this study was intention to screen for cervical cancer. This was measured using three questions: (i) If never screened for cervical cancer, would you like to be screened? With responses: ‘Yes,’ ‘No’ or ‘I do not know.’ ‘No’ and ‘I do not know’ were merged as No; (ii) If yes above, when do you intend to have the screening done? With responses: in three months, six months, one year, not sure and never; (iii) Where would you like to go for the screening? With responses: nearby government hospital, private health facility, regional referral hospital, or any other. A previous study in an area closer to the study area measured intention using two questions of whether one intended to go for screening and when [32], but we added a third question of where they intended to go for the screening. Those who responded ‘yes’ in the first question, intention to go in either three or six months or one year and indicated where they intended to go for screening were considered to have intentions to screen.

#### Independent variables

We measured cervical cancer knowledge using a 20-item scale consisting of four constructs: risk factors (six items); signs and symptoms (eight items); eligibility for screening (5 items); and routine cervical cancer screening recommendations (one item). A previous study in Eastern Uganda considered all women who scored above the average for 20-point possible answers to be more knowledgeable, while those who scored below the average were considered to have less knowledge [37]. For this study, we considered those who scored in the 75th percentile to be knowledgeable and those whose scores fell below the 75th percentile to be less knowledgeable. The other independent variables considered were age (categorized into 20–29, 30–39, 40–49 and 50–59); marital status (single, cohabiting/married, commercial Sex Worker/divorced/widowed); work status (employed, student/not-working); regular income (yes and no); highest level of education (A-level+, O-level, PLE, none); residence (rural area, town board, town council); family cancer history (don’t know, no, and yes); common source of health information (social media/FM radio, TV, CATs, health worker, and any other); perceived self-risk for cervical cancer (yes, no, I do not know); fear of getting diagnosed with cancer (yes, no, I do not know, refused to answer); and fear of the cervical cancer screening procedure (yes, no, I do not know, refused to answer).

### Statistical analysis

Descriptive statistics were reported using frequency distributions of the participant characteristics at individual level. Bivariate and multivariable analyses were conducted using generalized linear modelling with modified Poisson regression; prevalence ratios (PR) instead of odds ratios were used because of the high prevalence of intention to screen for cervical cancer [38, 39]. We built our final analytic model using backwards elimination, where only variables with a *p*-value ≤ 0.2 were considered for the adjustment stage to determine the factors independently associated with intention to screen for cervical cancer. Collinearity between independent variables was assessed using pairwise correlation analysis. Data were analysed using Stata/SE 17, and statistical significance was considered at *p* < 0.05; 95% confidence intervals are reported.

## Results

### Baseline characteristics of the participants

Half of the participants had intentions to be screened for cervical cancer. In addition, half the participants were aged 20–29 years, and nearly three-quarters (73%) were married or cohabiting, as shown in Table 1 below. There was almost an equal distribution in residence for rural, town board and town council. Three-quarters (76%) of the participants reported no family history of cervical cancer, and 46% considered themselves at risk of cervical cancer. Half (52.5%) of the participants had full-time jobs, 62% had a regular source of income, and only 12.8% had more than A-level (high school equivalent) education.

CATs were mentioned as the main source of health information for nearly half (49.8%); more than half (64%) also feared being diagnosed with cervical cancer, while 40.9% feared the screening procedure. Approximately one-quarter (26.5%) had moderate knowledge about cervical cancer.

**Table 1:** Baseline characteristics of adult women in Kyotera District, Central Uganda (*N* = 430)

Characteristic	Frequency (*n*)	Percentage (%)
**Intention to screen for cervical cancer**		
No Intention	214	49.77
Intention	216	50.23
Age category*		
20–29 years	215	50.00
30–39 years	126	29.30
40–49 years	62	14.42
50–59 years	27	6.28
**Marital Status**		
Single	72	16.74
Commercial Sex Worker/Divorced/Widowed	43	10.00
Cohabiting/Married	315	73.26
**Residence**		
Rural	150	34.88
Town board	141	32.79
Town council	139	32.33
**Family history of cervical cancer**		
No	329	76.51
Yes	39	9.07
Do not Know	62	14.42
**Working status**		
Full time	226	52.56
Part time	101	23.49
Not working	103	23.95
**Regular income**		
No	160	37.21
Yes	270	62.79
**Highest education level**		
Advanced level and above	55	12.79
Ordinary level	93	21.63
Primary Leaving Examination	156	36.28
None	126	29.30
**Primary source of health information**		
FM Radio/social media	130	30.50
Television	48	11.27
Community Audio Towers	212	49.78
Healthworker	36	8.45
**Consider self at risk of cervical cancer**		
No	115	27.78
Yes	194	46.86
Do not Know	105	25.36
**Fear of being diagnosed with cervical cancer**		
No	151	35.36
Yes	276	64.64
**Fear of cervical cancer screening procedure**		
No	251	59.06
Yes	174	40.94
**Knowledge about cervical cancer** (Mean = 2.5, SD = 2.0, Range = 0–11)		
Not knowledgeable	316	73.49
Knowledgeable	114	26.51

*Age was collected in categorical format, and we could not directly provide its distribution beyond this.

### Correlates of intention to screen for cervical cancer among adult women

At the bivariate level, knowledge of cervical cancer, residence, common source of health information and perceived risk of getting cervical cancer were associated with intention to screen for cervical cancer. After adjusting for potential confounders, only participants’ residence, common source of health information and perceived risk of getting cervical cancer were independently associated with intention to screen for cervical cancer. Participants who resided in the town council were 44% more likely to have intentions to screen for cervical cancer compared to those who lived in the rural areas (see Table 2).

Compared to participants who mentioned FM radio or social media as their main source of health information, those who mentioned television were 48% less likely to have intentions to screen for cervical cancer, while those who mentioned CATs/health workers were 36% less likely to have intentions to screen for cervical cancer. Participants who perceived themselves to be at risk of cervical cancer were 74% more likely to have intentions to screen compared to those who did not. The full results are presented in Table 2.

**Table 2:** Correlates of intention to screen for cervical cancer among adult women in Kyotera District, Central Uganda after adjustment

Variable	Intention^†^		cPR (95%CI)	*p* value	aPR (95%CI)	*p* value
	Yes f(c%)	No f(c%)				
**Age category**						
20–29 years	105(51.5)	105(53.8)	1		1	
30–39 years	62(30.4)	45(20.1)	1.16 (0.94–1.43)	0.171	1.09 (0.90–1.31)	0.386
40–49 years	28(13.7)	28(14.4)	1.00 (0.74–1.34)	1.000	1.02 (0.77–1.34)	0.902
50–59 years	9(4.4)	17(8.7)	0.69 (0.40–1.19)	0.187	0.79 (0.50–1.26)	0.326
**Marital Status**						
Single	29(14.2)	38(19.5)	1		1	
CSW/Divorced/Widow	18(8.8)	20(10.2)	1.09 (0.71–1.69)	0.683	0.96 (0.62–1.48)	0.852
Cohabiting/Married	157(77.0)	137(70.3)	1.23 (0.92–1.65)	0.162	1.07 (0.80–1.43)	0.623
**Residence**						
Rural	52(25.5)	81(41.5)	1		1	
Town board	72(35.3)	61(31.3)	1.38 (1.06–1.80)	0.016	1.18 (0.93–1.49)	0.164
Town council	80(39.2)	53(27.2)	1.54 (1.19–1.98)	0.001*	1.44 (1.12–1.86)	0.004*
**Family History of cervical cancer**						
No	161(78.9)	142(72.8)	1		1	
Yes	21(10.3)	16(8.2)	1.07 (0.79–1.44)	0.668	0.87 (0.65–1.16)	0.333
Do not Know	22(20.8)	37(19.0)	0.70 (0.49–0.99)	0.046	0.89 (0.69–1.15)	0.376
**Highest Education level**						
A’ level and above	24(11.8)	24(12.3)	1		1	
O’ level	36(17.6)	49(25.1)	0.85 (0.58–1.23)	0.388	0.95 (0.67–1.35)	0.785
PLE	72(35.3)	78(40.0)	0.96 (0.69–1.33)	0.808	1.06 (0.77–1.46)	0.717
None	72(35.3)	44(22.6)	1.24 (0.90–1.70)	0.181	1.23 (0.91–1.66)	0.183
**Common source of health information**						
FM Radio/social media	86(42.4)	32(16.7)	1		1	
TV	16(7.9)	31(16.1)	0.47 (0.31–0.71)	< 0.001**	0.52 (0.34–0.82)	0.005*
CATs	89(43.8)	106(55.2)	0.63 (0.52–0.76)	< 0.001**	0.64 (0.52–0.80)	< 0.001**
Health-worker	12(5.9)	23(12.0)	0.47 (0.29–0.75)	0.002*	0.64 (0.43–0.98)	0.040
**Perceived risk of getting cervical cancer**						
No	34(16.7)	73(40.8)	1		1	
Yes	118(57.8)	62(34.6)	2.06 (1.53–2.78)	< 0.001**	1.74 (1.28–2.36)	< 0.001**
Do not Know	52(25.5)	44(24.6)	1.70 (1.2–2.38)	0.002*	1.52 (1.09–2.12)	0.013
**Fears being diagnosed with Cervical cancer**						
No	65(31.9)	80(41.7)	1		1	
Yes	139(68.1)	112(58.3)	1.23 (0.99–1.53)	0.051	1.09 (0.89–1.33)	0.406
**Knowledge on cervical cancer**						
Less knowledgeable	139(46.2)	162(53.8)	1		1	
Knowledgeable	65(66.3)	33(33.7)	1.43 (1.19–1.73)	< 0.001**	1.17(0.96–1.42)	0.122

**cPR**: crude prevalence ratio; **aPR**: adjusted prevalence ratio.

^**†**^Column totals differ due to differences in responses to different variable specific questions

## Discussion

This study assessed the correlates of intention to screen for cervical cancer in Kyotera district, Central Uganda. We found 50.2% of the participants had intentions of being screened for cervical cancer. This prevalence was slightly lower than the 63% reported in a neighbouring district of Masaka in 2013 [32] and the 61% reported in rural Indonesia in 2016 [40]. However, it is higher than the prevalence of 45.3% in Ethiopia in 2017 [41]. Given the length of time since these prior estimates, we classify the prevalence of intention to screen in our study as suboptimal; we would anticipate increasing rates of screening over time and that there is a gap in cervical cancer related health promotion. As such, additional efforts are needed to promote cervical cancer screening in this area.

Participants who resided in the town council were 44% more likely to have intentions to screen for cervical cancer compared to those who lived in rural areas. This could be attributed to geographic proximity to health services located in urban areas, as well as the potential differences in income between urban and rural areas. Women who lived in urban and semiurban areas in Eastern Uganda were four times and two times more likely to have high knowledge about cervical cancer than their rural counterparts, respectively [37]. Our findings and others indicate there is a disparity in intentions to screen, which likely translates to differences in screening uptake. More equitable approaches to service delivery are warranted, including increased funding to support health education and cervical cancer screening promotion.

CATs were mentioned as the main source of health information for 49.8% of participants, compared to only 8.4% reported health workers as their main source of health information. The proportion that reported health workers was lower than the 15.1% reported from health facilities in Eastern Uganda [37]. Another study conducted in a neighbouring district had found that women who had discussions on cervical cancer with health care providers reported more intentions to screen for cervical cancer [32]. Even among women in Thailand, having received a recommendation from health care providers was associated with decisions to attend cervical cancer screening [42]. As such, integrating cervical cancer screening into health workers education packages and disseminating information via CATs may be effective health communication delivery mechanisms, where available. This may be feasible in Kyotera since a previous study found that the majority of the health workers believed CATs were accessible and easier to communicate on health issues; however, fewer than 20% used them [43].

We found only 46% of the sample considered themselves at risk of cervical cancer compared to 76.0% who perceived themselves to be at risk of cervical cancer in another study in Eastern Uganda, as reported in 2017 [44]. Risk perceptions were identified to be particularly important since those who perceived themselves to be at risk of cervical cancer were 74% more likely to have intentions to screen compared to those who did not; these findings align with prior reports in a neighbouring district [32]. Relatedly, a family history of cervical cancer was not associated with higher intentions of screening in this study, but it was reported to be associated among women in rural areas of Indonesia [40]; further research is needed to identify potential differences between family history and the impact on cancer screening. Multiple approaches for conducting effective Health education should be strengthened including use of print and interpersonal communication, as this could to help increase risk perception, increase intentions for screening, and ultimately aid in increasing uptake of cervical cancer screening.

Although being knowledgeable about cervical cancer was not associated with intentions to screen for cervical cancer after adjustment, it is important to note that only 26.5% of the participants had moderate knowledge (> 75th percentile) about cervical cancer. Increasing knowledge about cervical cancer is a critical area for further intervention given its importance in decision-making; yet it is likely this factor significant at the bivariate level was no longer significant after adjustment because of potential correlation with other social determinants of health (e.g., rurality). Knowledge has been reported to be associated with intention to undergo Pap smear testing in rural areas of Indonesia [40]. Therefore, improving knowledge about cervical cancer literacy could improve screening uptake.

Over half of the participants feared being diagnosed with cervical cancer, while 40.9% feared the screening procedure. Although these fears were not associated with intentions to screen for cervical cancer after adjustment, they could remain potential barriers to screening. These findings are inconsistent with previous studies conducting in neighbouring districts, Thailand, and Ethiopia [32, 42, 45]. Additional qualitative research could help identify nuance in these reports and is recommended; decreasing barriers to screening and managing a diagnosis are important to support patients in cancer prevention.

### Strengths and limitations

We applied approaches to maximize the validity of the findings of this study. First, we assessed the outcome variable with more than a single question to only consider those who indicated the intention as well as when and where they would go for screening as those with intention to minimize social desirability bias. The district-wide sampling and the rich distribution of participants by age are other strengths of this study and are key to representativeness and thus generalizability of the study findings across the district and similar contexts. Despite these, some limitations are acknowledged. First, there could have been some people who still indicated intentions without actual intentions. In addition, the inherent limitations of cross-sectional study design including recall and difficulties with self-reporting on other variables other than intention cannot miss acknowledgement.

## Conclusion

In this study, we found only half of adult women sampled in the Kyotera district, Central Uganda, had intentions for cervical cancer screening, and only 46% considered themselves at risk of cervical cancer. Urban residence, risk perception, and CATs as a source of health information were associated with higher intentions to screen for cervical cancer. The urban-rural difference calls for equity in cervical cancer health education and service delivery. In addition to other communication channels, targeting health information sharing via CATS and interactive TV educational messages may help reach those with lower intentions to screen.

## Data Availability

The datasets used and/or analysed during the current study available from the corresponding author on reasonable request.
