# Correction: Correlates of intention to screen for cervical cancer among adult women in Kyotera District, Central Uganda: a community based cross-sectional study

**DOI:** 10.1186/s12905-024-03155-3

**Published:** 2024-06-06

**Authors:** Richard Kabanda, Arthur Kiconco, Anguzu Ronald, Kirsten M. M. Beyer, Steven A. John

**Affiliations:** 1 Institute for Health and Equity, Medical College of Wisconsin, Milwaukee, WI 53226 USA; 2 Uganda Martyrs University, Nkozi, Uganda; 3 Ministry of Health, Kampala, Uganda; 4 Department of Psychiatry and Behavioral Medicine, Medical College of Wisconsin, Milwaukee, WI 53226 USA

**Correction to: BMC Women’s Health (2024) 24:296**

10.1186/s12905-024-03129-5

In this article [1], the order of the author’s list has been updated.

The original article has been corrected.
