# Correction: Correlates of intention to screen for cervical cancer among adult women in Kyotera District, Central Uganda: a community based cross-sectional study

**DOI:** 10.1186/s12905-024-03500-6

**Published:** 2024-12-21

**Authors:** Arthur Kiconco, Richard Kabanda, Anguzu Ronald, Kirsten M. M. Beyer, Steven A. John

**Affiliations:** 1 Institute for Health and Equity, Medical College of Wisconsin, Milwaukee, WI 53226 USA; 2 Uganda Martyrs University, Nkozi, Uganda; 3 Ministry of Health, Kampala, Uganda; 4 Department of Psychiatry and Behavioral Medicine, Medical College of Wisconsin, Milwaukee, WI 53226 USA

**Correction** **:** **BMC Women’s Health 24, 296 (2024)**

**https://doi.org/10.1186/s12905-024-03129-5**

The original publication of this article [1], the order of the authors appeared in the author list was incorrect. Arthur Kiconco should be listed as the first author and Richard Kabanda as the second author.

Incorrect Author group

Richard Kabanda^1*^, Arthur Kiconco^2,3^, Anguzu Ronald^1^, Kirsten M. M. Beyer^1^ and Steven A. John^4^

Correct Author group

Arthur Kiconco^1^, Richard Kabanda^2,3*^, Anguzu Ronald^1^, Kirsten M. M. Beyer^1^ and Steven A. John^4^

The original article has been corrected.
